# Objective Assessment of Hyposmia in Alzheimer's Disease From Image and Behavior by Combining Pleasant Odor With Unpleasant Odor

**DOI:** 10.3389/fneur.2021.697487

**Published:** 2021-09-06

**Authors:** Quanzhi Feng, Hui Liu, Hui Zhang, Yi Liu, Huihong Zhang, Yuying Zhou, Gang Liu, Tong Han

**Affiliations:** ^1^Department of Radiology, Tianjin Huanhu Hospital, Tianjin University, Tianjin, China; ^2^Tianjin Key Laboratory of Cerebral Vascular and Neurodegenerative Diseases, Tianjin, China; ^3^Department of Radiology, First Teaching Hospital of Tianjin University of Traditional Chinese, Tianjin, China; ^4^Department of Ultrasound, Tianjin Huanhu Hospital, Tianjin, China; ^5^Department of Neurology, Tianjin Huanhu Hospital, Tianjin, China; ^6^Department of Otolaryngology, Tianjin Huanhu Hospital, Tianjin, China

**Keywords:** olfaction, functional magnetic resonance imaging, primary olfactory cortex, Alzheimer's disease, odor

## Abstract

**Background:** Olfactory functional magnetic resonance imaging (fMRI) of responses to a pleasant odor (PO) (lavender) can objectively evaluate olfactory dysfunction in Alzheimer's disease (AD) patients. The brain responses to a PO and unpleasant odor (UPO) were shown to differ in normal young people. Whether AD patients with olfactory dysfunction have the same brain response is not yet known.

**Objective:** Our aim was to explore whether olfactory fMRI with both a PO and UPO can provide more information regarding olfactory impairment in AD than a PO alone.

**Methods:** Twenty-five normal controls (NC), 26 individuals with mild cognitive impairment (MCI), and 22 AD patients underwent olfactory fMRI with lavender and pyridine odorants at three concentrations (0.10, 0.33, and 1.00%) with a 3.0-T MRI scanner.

**Results:** There were no differences in the number of activated voxels in the primary olfactory cortex (POC) between PO and UPO conditions in the NC, MCI, and AD groups (SPM, paired *t*-test, uncorrected *p* < 0.001, extent threshold = 70). In the right inferior frontal gyrus, orbital part (F3O), the number of activated voxels was greater with the UPO than with the PO in the NC group (SPM, paired *t*-test, uncorrected *p* < 0.001, extent threshold = 70), but there were no differences in the MCI and AD groups. Regardless of PO or UPO conditions, there were significant differences in the number of activated voxels in the POC among the NC, MCI, and AD groups. With increasing odor concentration, the number of activated voxels in the POC decreased in the NC group but increased in the AD group. When 0.10% UPO was presented, the NC group (21/25) showed a lower breathing amplitude and shorter inhalation time, whereas the AD patients (0/22) did not show such changes in breathing.

**Conclusions:** After PO and UPO inhalation, brain activation and respiratory behavior in AD patients were significantly different than in NC patients. Therefore, olfactory fMRI using both PO and UPO stimulation, compared with PO stimulation only, can provide more objective information regarding hyposmia associated with AD based on imaging and behavior.

## Introduction

It is a well-established fact that Alzheimer's disease (AD) is the most common dementia and is subdivided into typical and atypical AD (1). Typical AD mainly manifests as memory dysfunction, and atypical AD includes posterior cortex atrophy, a logopenic variant of primary progressive aphasia, and a frontal variant of AD. Studies (2–4) have shown that olfactory disorder is one of the early clinical manifestations and may be one of the important markers for screening early typical AD (5, 6). Commonly used clinical methods for detecting olfactory function include the University of Pennsylvania Smell Identification Test (7–10), Sniffin' Sticks (11–13), T&T (14–16), and so on, which are relatively easy to use but obviously subjective. However, functional magnetic resonance imaging (fMRI), as a new imaging method, has been shown to objectively detect olfactory function (17). Olfactory fMRI studies using a pleasant odor (PO) (lavender) in normal elderly, individuals with mild cognitive impairment (MCI), and AD patients by Zhang et al. (18) found significant differences in the primary olfactory cortex (POC), with significantly more activated voxels in normal elderly individuals than in MCI and AD patients. Meanwhile, the activation patterns in the POC showed that normal elderly individuals had olfactory adaptation, but AD patients did not. In other words, olfactory fMRI reflected impairments in olfactory function caused by pathological changes in AD.

Previous olfactory fMRI studies have shown that the POC (19) and orbitofrontal cortex (20, 21) are involved in olfactory identification. There were significant differences in the activation of the above brain regions in normal young people when a PO and an unpleasant odor (UPO) were inhaled. The activation caused by the UPO was more significant than that caused by the PO. There is a lack of reliable research evidence that explores whether AD patients with olfactory dysfunction have the same brain responses. Previous studies (17, 18, 22, 23) using olfactory fMRI with AD patients have used PO (lavender), but there has been no published evidence of brain activation with a UPO in AD patients with olfactory impairment. Because of pathological depositions (neurofibrillary tangles and senile plaques) deposited in the POC (24, 25), olfactory function, including olfactory perception and identification ability, of AD patients is impaired in the early stages (26, 27). With the progression of disease, there are extreme situations where fragrances and stenches cannot be distinguished, which indicates that the patient's olfactory identification ability is seriously damaged, and perhaps, the function is lost. Therefore, we hypothesized that there may be “less difference” or even “no difference” in the relevant brain regions involved in olfactory identification (such as the POC and orbitofrontal cortex) when comparing the PO and UPO fMRI results due to the impaired olfactory identification ability of AD patients.

In addition, normal people easily accept smelling a PO, which does not affect respiratory behavior, whereas smelling a UPO results in resistance and subconscious respiratory adjustments. The difference in breathing behavior of normal people after inhaling a PO or UPO reflects normal olfactory identification function. In AD patients, because of impaired olfactory function, the ability to distinguish PO and UPO may decrease. To prove this point, we hypothesized that breathing behavior in AD patients might differ from that in normal controls (NCs) when inhaling a PO and UPO.

In this study, NC, MCI, and AD subjects underwent PO and UPO fMRI to confirm our hypothesis and to explore whether the combination of PO and UPO fMRI, compared with PO alone, can provide more objective information regarding AD patients with olfactory impairment.

## Materials and Methods

### Subjects

This study was approved by the local institutional review board, and all patients signed informed consent forms. The MCI and AD patients included in this experiment were treated in the outpatient clinic of a single-center hospital from July 2015 to December 2016. All subjects were subjected to standardized neuropsychological tests that included the Mini-Mental State Examination (MMSE) and Montreal Cognitive Assessment (MoCA), Clinical Dementia Rating (CDR) scale assessment, and associated history collection. AD patients met the typical AD diagnostic criteria in the new AD diagnostic criteria [the international working group (IWG-2) criteria] (1). Based on Peterson criteria (28), the inclusion criteria for patients with amnestic MCI were as follows: (1) complaints of decreased memory; (2) MMSE score >24 points, MoCA score <26 points, and CDR score of 0.5. The exclusion criteria were as follows: (1) other central and peripheral diseases affecting olfaction, namely, brain trauma, cerebrovascular disease, schizophrenia, Parkinson disease, depression, psychotropic drug users, active sinusitis, smokers, and respiratory tract obstruction; (2) contraindications against or claustrophobia with the MRI examination; and (3) failure to cooperate or poor MRI image quality. These subjects were selected by neurology experts with 20 years of working experience based on the above criteria.

A total of 99 subjects were enrolled, all right-handed, including 11 individuals with other central and peripheral diseases affecting olfaction, 2 individuals with claustrophobia, and 13 individuals who failed to cooperate during the examination or had poor image quality. Finally, 73 subjects were enrolled, including those assigned to the NC group (*n* = 25, 67.10 ± 6.53 years), MCI group (*n* = 26, 67.04 ± 6.95 years), and AD group (*n* = 22, 68.41 ± 6.61 years).

### Olfactory fMRI Design

An olfactory stimulator (Emerging Tech Trans, LLC, Hershey, PA, USA) was used to present odorants and to monitor and record the breathing movements of subjects. It mainly includes three parts: (1) the main chassis includes file management, experimental mode setting, and manual controls; (2) a pipeline (Teflon hose) and multichannel valve control system, in which the flow rate is controlled at 6 L/min, and the gas in the pipeline can be quickly delivered and removed; (3) the odor sources, in particular, six groups of odor sources, were placed at the end of the stimulator pipeline.

The odorants included lavender oil (17) and pyridine (29). Lavender oil is a PO, diluted by 1,2-propanediol to 0.10, 0.33, and 1.00% concentrations; pyridine is a UPO, diluted by distilled water to the same concentrations as the PO; both odorants are volatile liquid preparations. These odorants can cause significant olfactory central nervous system activity, and a small amount of inhalation has no toxic side effects on the subjects.

**Figure 1:**
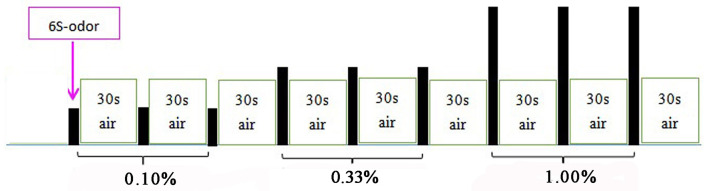
Olfactory stimulation mode. Each odor lasted for 6 s, followed by 30 s of clean odorless air; 0.10, 0.33, and 1.00% odors were used in the first three, second three, and third three stimulus, respectively. PO was presented first; UPO was presented after a rest for 25 min.

We conducted two olfactory stimulation tasks. The first task involved the delivery of lavender, and 25 min later, the second task involved the delivery of pyridine. Each olfactory stimulation task included an initial 6-s delivery of the odorant followed by 30 s of clean odorless air. Each concentration was presented three times in succession, beginning with the weakest concentration (Figure 1). Before the experiment, the subjects were not trained to be familiar with the stimulus paradigm but were told only that they would smell the odor during the scanning process and instructed to keep their head and body motionless during the whole examination process. During the two stimulation tasks, the image acquisition and odor presentation were synchronized, and the subjects' breathing process was recorded.

### Imaging Protocol

The imaging data were acquired on a Siemens Trio Tim 3.0-T magnetic resonance scanner with a 12-channel head coil. A blood oxygen level–dependent (BOLD) signal-sensitive T2^*^-weighted echo planar imaging sequence was used to acquire functional data with the following parameters: slice number = 36, repetition time (TR)/echo time (TE)/flip angle (FA) = 2,000 ms/30 ms/90°, field of view (FOV) = 230 × 230 mm, matrix = 64 × 64 mm, slice thickness = 4 mm, and acquisition time (TA) = 6 min 2 s. T1-weighted images with 1-mm isotropic resolution were acquired with the magnetization-prepared rapid acquisition gradient echo (MPRAGE) method: TR/TE/FA = 2,000 ms/2.26 ms/9°, FOV = 256 × 256 mm, matrix = 256 × 256 mm, slice number = 192, voxel size = 1 × 1 × 1 mm, and TA = 6 min 48 s.

### fMRI Data Processing

**Figure 2:**
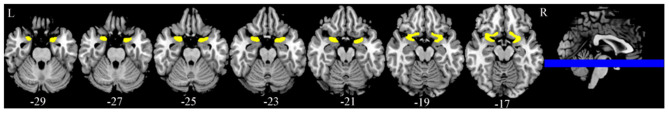
Bilateral primary olfactory cortex (POC). The central MNI coordinates are (−24, 0, −17) and (24, 0, −17).

**Figure 3:**
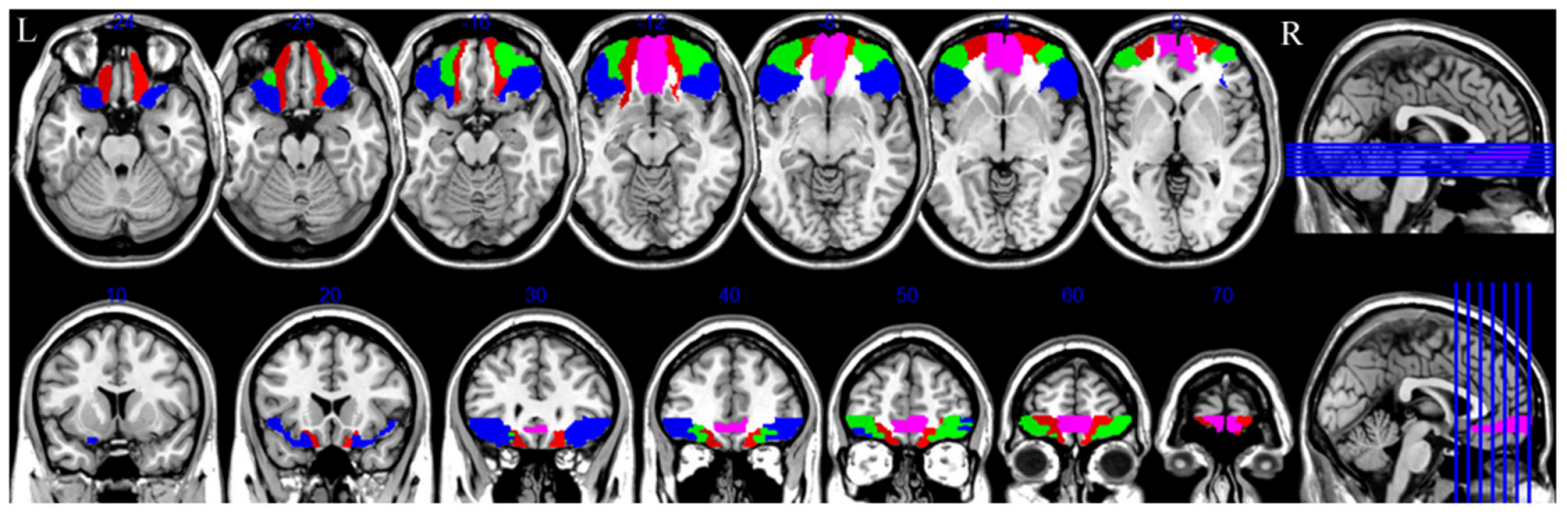
Bilateral orbitofrontal cortex. The red part is superior frontal gyrus, orbital part; the pink part is superior frontal gyrus, medial orbital; the green part is middle frontal gyrus, orbital part; the blue part is inferior frontal gyrus, orbital part. R, right.

Imaging data were analyzed utilizing Statistical Parametric Mapping (SPM8, Wellcome Trust Centre for Neuroimaging, University College London, UK). The first 10 images of each fMRI raw data set were discarded to remove the initial transit signal fluctuations. The subsequent images were processed: slice timing; realignment (subjects with over 1.5 mm panning in 3D and rotation of over 1.5 degrees were excluded), normalization (functional images with voxels of 3 × 3 × 4 mm and T1-weighted high-resolution anatomical images with voxels of 1 × 1 × 1 mm were registered, and then structural images after registration were segmented and normalized into standard space), and Gaussian smoothing (full width at half maximum was [8 8 8]). An SPM was generated for each subject under each odorant concentration condition by fitting the stimulation paradigm to the functional data convolved with a hemodynamic response function and time derivative. The voxels of the active structures were overlaid on the 3D T1-weighted anatomical image in Montreal Neurological Institute (MNI) coordinates. The POC and orbitofrontal cortex were selected as regions of interest (ROIs). Based on the method of Wang et al. (17) and Vasavada et al. (22), the POC (Figure 2) was outlined, including the anterior olfactory nucleus, olfactory tubercle, piriform cortex, anterior portion of the periamygdaloid cortex and amygdala, and anterior perforated substance. In the standard MNI space, the central MNI coordinates of the bilateral POC were (−24, 0, −17) and (24, 0, −17). As the two odors were paired and compared within the same individuals, the individual differences in the POC were eliminated. The orbitofrontal cortex included the following regions extracted from the automated anatomical labeling template (30): the superior frontal gyrus, orbital part (F1O); superior frontal gyrus, medial orbital part (F1MO); middle frontal gyrus, orbital part (F2O); and inferior frontal gyrus, orbital part (F3O) (Figure 3).

### Breathing Behavior of Subjects Recorded by the Olfactory Stimulator

**Figure 4:**
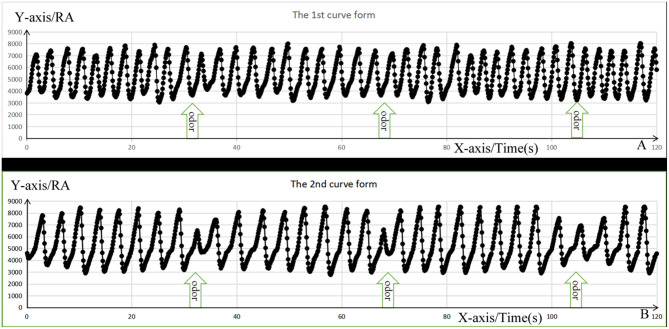
The change form of respiratory amplitude curve. The *X*-axis represents respiratory time; the *Y*-axis represents respiratory amplitude (RA) value, and the black dots represent the values of RA per ms. **(A)** Represents the first respiratory form with no significant respiratory change, whereas **(B)** represents the second respiratory form with short ventilation during inspiration, followed by decrease in the breathing amplitude, shortening the inhalation time, and gradually getting their breath back.

The respiratory results recorded by the olfactory stimulator were copied to the computer, the respiratory amplitude value per millisecond of exposure to the two odors was extracted, and the curves for respiratory amplitude were drawn, which can be summarized as follows: (1) there was no obvious respiratory change (Figure 4A); (2) there were obvious respiratory changes that included an inspiratory process during odor stimulation or the first inspiratory process after the stimulation that involved a short ventilation period followed by a significant decrease in the breathing amplitude, shortened inhalation time, and gradual return to normal (Figure 4B). The changes in the respiratory amplitude curves among the three groups are listed in Table 1.

**Table 1:** The respiratory amplitude curve form of subjects among three groups under PO and UPO.

**Odor type**	**Group**	**The 1st curve form, n**	**The 2nd curve form, *N* (low concentration stimulation)**	**The 2nd curve form, *N* (moderate and high concentration stimulation)**	***χ^2^***	***p***
PO	NC	20	0	5	5.108^#^	0.074
	MCI	22	0	4		
	AD	22	0	0		
UPO	NC	4	17	4	31.275	0.000^*^
	MCI	4	9	13		
	AD	14	0	8		

*PO, pleasant odor; UPO, unpleasant odor; NC, normal control; MCI, mild cognitive impairment; AD, Alzheimer's disease*.

# *Fisher exactly test*.

* *p < 0.05*.

### Statistical Analysis

The statistical software SPSS 19.0 and SPM8 were used. One-way analysis of variance was used to compare demographic information and neurocognitive test results (*p* < 0.05). The ROI activations during olfactory fMRI under PO and UPO conditions were compared using SPM (paired *t*-test, uncorrected *p* < 0.001, extent threshold = 70) and Kruskal–Wallis rank sum test (*p* < 0.05) followed by Bonferroni *post-hoc* tests among the three groups. Regression analysis was used to confirm the correlation between the number of activated voxels in the ROIs and the MMSE and MoCA scores. We used χ^2^-tests to assess the differences in sex and breathing behavior among the three groups (*p* < 0.05).

## Results

### Demographics of the Participants

The demographic information and neurocognitive test (MMSE and MoCA) results for the three groups are shown in Table 2. There were no differences between the patient groups and NCs in age, sex, or education level. However, as expected, the MCI and AD patients scored significantly lower than the NCs on the MMSE and MoCA.

**Table 2:** Demographic information and neurocognitive tests.

**Group**	***n***	**Gender (M/F)**	**Age (y)**	**Education**	**MMSE**	**MoCA**
NC	25	10/15	67.10 ± 6.53	10.38 ± 3.56	28.48 ± 1.40	26.95 ± 1.83
MCI	26	9/17	67.04 ± 6.95	10.81 ± 3.14	28.00 ± 12.79	21.81 ± 2.87
AD	22	9/13	68.41 ± 6.61	10.32 ± 3.55	17.23 ± 5.96	12.91 ± 5.85
*F*		0.09^a^	0.31	0.15	55.71	72.80
*p*		0.91	0.74	0.86	0.000^*^	0.000^*^

*NC, normal control; MCI, mild cognitive impairment; AD, Alzheimer's disease*.

a *Represents the χ^2^-value*.

* *p < 0.05*.

### ROI Activation

**Figure 5:**
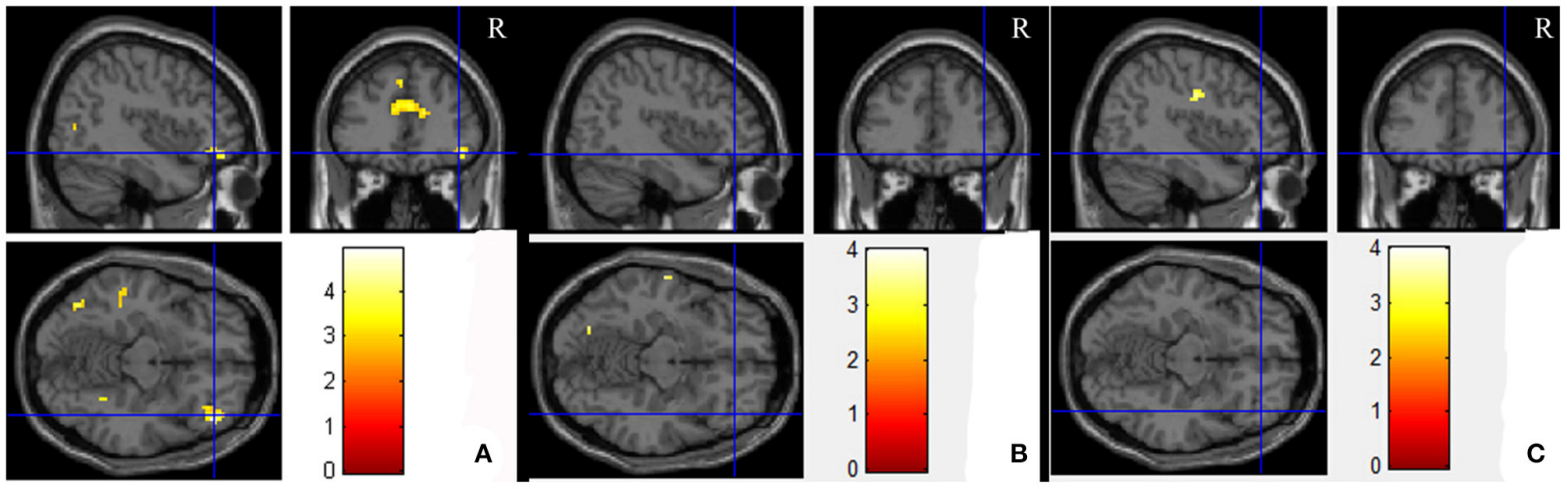
The average brain activation map for the UPO higher than PO in the NC, MCI, and AD groups (paired *t*-test, uncorrected *p* < 0.001, extent threshold = 70). **(A–C)** Are NC, MCI, and AD, respectively.

Paired *t*-tests for the comparison between the PO and UPO showed that there were no differences in the number of activated voxels in the POC in the NC group, MCI group, and AD group (SPM, uncorrected *p* < 0.001, extent threshold = 70). In the orbitofrontal cortex, there was a significant difference in the number of activated voxels in the right F3O in the NC group, the number of activated voxels in the UPO group was greater than that in the PO group, and there were no significant differences between groups in the F1O, F1MO, and F2O (SPM, paired *t*-test, uncorrected *p* < 0.001, extent threshold = 70) (Figure 5A). In the MCI group and AD group, F1O, F1MO, F2O, and F3O showed no significant differences (Figures 5B,C). Table 3 provides a summary of some activated cluster locations and sizes in the NC group.

**Table 3:** Brain activated regions responding to the UPO higher than PO in NC.

**Area**	**MNI coordinates**			**Activated size (voxels)**	***t*-value**
	***X***	***Y***	***Z***		
Cingulum_anterior_R	9	21	21	154	7.36
Occipital lobe_R	15	−78	3	126	6.55
Angular_L	−48	−57	30	94	6.18
Precuneus_R	3	−66	30	90	6.11
Frontal inferior orbit_R	−45	−18	−6	80	5.94
Middle temporal gyrus_L	−51	−36	−9	80	5.88
Superior frontal gyrus_L	−18	24	48	71	5.36

*PO, pleasant odor; UPO, unpleasant odor; NC, normal control; R, right; L, left*.

*t-value, paired t-test (SPM, p < 0.001, uncorrected, extent threshold = 70)*.

### POC Activation Under Low, Medium, and High Concentrations

Table 4 shows the number of activated voxels in the POC under three concentrations of the PO and UPO among the three groups. The intergroup comparison showed that there were differences in the number of activated voxels among the NC, MCI, and AD groups at 0.10, 0.33, and 1.00% concentrations, regardless of whether the PO or UPO was used (PO: *p* = 0.000, 0.000, 0.028; UPO: *p* = 0.000, 0.000, 0.033, respectively).

**Table 4:** Comparison of activated voxel of primary olfactory cortex (POC) in three groups under two odors.

**Odor type**	**Concentration**	**NC (*n* = 25)**	**MCI (*n* = 26)**	**AD (*n* = 22)**	***H***	***p***
		**Activation voxel numbers [P**_****50****_ **(P**_****25****_ **– P**_****75****_ **)]**				
PO	0.10%	23.00 (3.50–58.50)^∧^^*^	2.50 (0.00–14.25)^*^	0.00 (0.00–0.00)^∧^	20.427	0.000
	0.33%	33.00 (5.00–66.50)^∧^^*^	0.50 (0.00–40.75)^*^	0.00 (0.00–0.00)^∧^	19.314	0.000
	1.00%	21.00 (0.00–45.00)	9.00 (0.00–47.50)^#^	0.00 (0.00–19.25)^#^	7.139	0.028
UPO	0.10%	42.00 (2.50–86.50)^∧^^*^	4.00 (0.00–21.50)^*^	0.00 (0.00–0.00)^∧^	20.930	0.000
	0.33%	35.00 (3.00–63.00)^∧^	15.50 (0.00–37.50)^#^	0.00 (0.00–10.5)^∧^^#^	15.266	0.000
	1.00%	15.00 (0.00–39.00)^∧^	2.50 (0.00–64.25)	0.00 (0.00–21.25)^∧^	6.818	0.033

*PO, pleasant odor; UPO, unpleasant odor; NC, normal control; MCI, mild cognitive impairment; AD, Alzheimer's disease; H, Kruskal–Wallis rank sum test*.

*Bonferroni post-hoc tests among the three groups*.

* *Significant difference between NC and MCI groups*.

# *Significant difference between MCI and AD groups*.

∧ *Significant difference between NC and AD groups*.

Using Bonferroni *post-hoc* tests to correct the significance level, the following comparisons were made: at the 0.10% concentration of the PO, the NC and MCI groups (*p* = 0.038) and the NC and AD groups (*p* = 0.000) were different, and the MCI and AD groups (*p* = 0.098) showed no difference; at the 0.33% concentration of the PO, the NC and MCI groups (*p* = 0.038) and the NC and AD groups (*p* = 0.000) were different, and the MCI and AD groups (*p* = 0.134) showed no difference; and at the 1.00% concentration of the PO, the NC and MCI groups (*P* = 1.000) and the NC and AD groups (*p* = 0.070) showed no difference, and the MCI and AD groups (*p* = 0.048) were different.

At the 0.10% concentration of the UPO, the NC and MCI groups (*p* = 0.045) and the NC and AD groups (*p* = 0.000) were different, and the MCI and AD group (*p* = 0.072) showed no difference; at the 0.33% concentration of the UPO, there were differences between the MCI and AD groups (*p* = 0.038) and the NC and AD groups (*p* = 0.000) with no difference between the NC and MCI groups (*p* = 0.425); and at the 1.00% concentration of the UPO, there were no differences between the NC and MCI groups (*p* = 1.000) and the MCI and AD groups (*p* = 0.137), and there was a difference between the NC and AD groups (*p* = 0.038).

The intragroup comparisons showed that with the gradual increase in PO concentration, the number of activated voxels in the NC group first increased and then decreased, and the number of activated voxels in the MCI group and AD group gradually increased; with the gradual increase in UPO concentration, the number of activated voxels in the NC group gradually decreased, and the number of activated voxels in the MCI group and AD group gradually increased.

### Correlations Between Neurocognitive Test Results and the Number of Activated Voxels in the ROI

#### Correlations Between the Number of Activated Voxels in the POC and the MMSE and MoCA Scores

**Figure 6:**
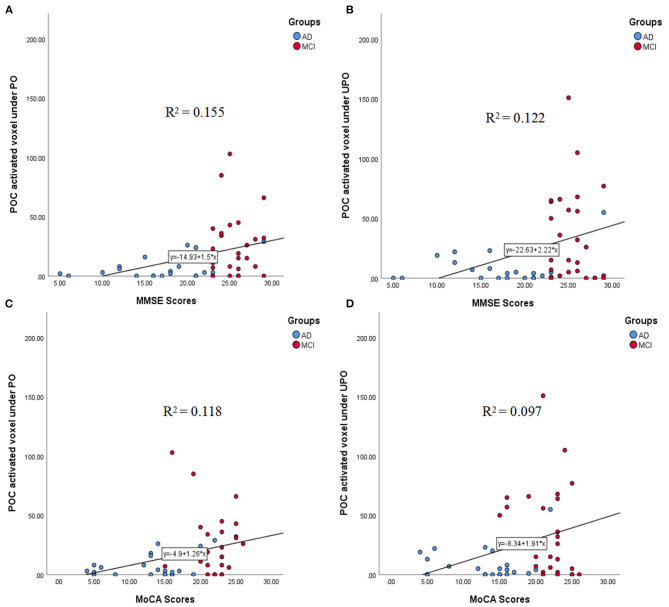
**(A–D)** The correlations between the number of activated voxels in the POC and the MMSE and MoCA scores in the PO and UPO conditions. **(A)** There was a significant correlation between the number of POC activated voxels and the MMSE scores under PO (*r* = 0.394, *p* = 0.005). **(B)** There was a significant correlation between the number of POC activated voxels and the MMSE scores under UPO (*r* = 0.350, *p* = 0.014). **(C)** There was a significant correlation between the number of POC activated voxel and the MoCA scores under PO (*r* = 0.344, *p* = 0.015). **(D)** There was a significant correlation between the number of POC activated voxel and the MoCA scores under UPO (*r* = 0.311, *p* = 0.030).

The correlations between the number of activated voxels in the POC and the MMSE and MoCA scores in the PO and UPO conditions are shown in Figures 6A–D. The number of activated voxels in the POC was correlated with MMSE scores (PO: *r* = 0.394, *p* = 0.005; UPO: *r* = 0.350, *p* = 0.014). The number of voxels was related to MoCA scores (PO: *r* = 0.344, *p* = 0.015; UPO: *r* = 0.311, *p* = 0.030). MMSE scores in the AD group mainly ranged from 10 to 20, and MoCA scores mainly ranged from 8 to 20.

#### Correlations Between the Number of Activated Voxels in the Right F3O and the MMSE and MoCA Scores

**Figure 7:**
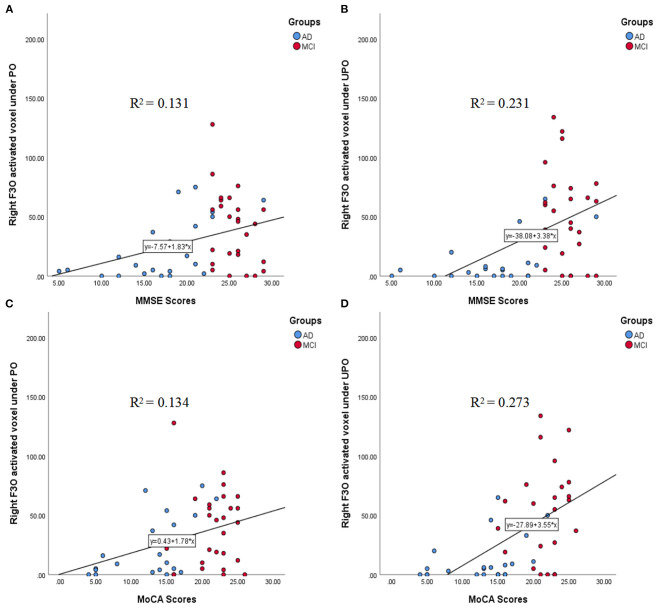
**(A–D)** The correlations between the number of activated voxels in the right F3O and the MMSE and MoCA scores in the PO and UPO conditions. **(A)** There was a significant correlation between the number of right F3O activated voxels and the MMSE scores under PO (*r* = 0.363, *p* = 0.010). **(B)** There was a significant correlation between the number of right F3O activated voxels and the MMSE scores under UPO (*r* = 0.480, *p* = 0.000). **(C)** There was a significant correlation between the number of right F3O activated voxels and the MoCA scores under PO (*r* = 0.298, *p* = 0.037). **(D)** There was a significant correlation between the number of right F3O activated voxels and the MoCA scores under UPO (*r* = 0.460, *p* = 0.001).

The correlations between the number of activated voxels in the right F3O and the MMSE and MoCA scores in the PO and UPO conditions are shown in Figures 7A–D. The number of activated voxels in the right F3O was correlated with MMSE scores (PO: *r* = 0.363, *p* = 0.010; UPO: *r* = 0.480, *p* = 0.000). The number of voxels was related to MoCA scores (PO: *r* = 0.298, *p* = 0.037; UPO: *r* = 0.460, *p* = 0.001).

### Comparison of Respiratory Behavior in the PO and UPO Conditions Among the Three Groups

When the PO was presented, most subjects in the NC and MCI groups had no significant respiratory behavior changes, with the exception of a few subjects who had respiratory behavior changes at medium and high concentrations (0.33 and 1.00%); all subjects in the AD group did not show significant respiratory behavior changes at the three concentrations. Most of the subjects in the NC and MCI groups changed their respiratory behavior when they were stimulated by the UPO. The difference was that most of the subjects in the NC group changed their respiratory behavior at low concentrations (0.10%), whereas most of the subjects in the MCI group showed similar behavior changes at medium and high concentrations. Approximately two-thirds of the subjects in the AD group did not change their respiratory behavior at any of the three concentrations, and one-third of the subjects showed respiratory behavior changes at the 0.33 and 1.00% concentrations.

Comparisons of the respiratory behavior of subjects among the three groups were performed with χ^2^-tests. As there were many cells with a theoretical frequency of <1 during PO stimulation (Table 1), the respiratory behavior with PO stimulation was tested using Fisher exact test. The respiratory behavior of the three groups under PO stimulation showed no significant difference (χ^2^ = 5.108, *p* = 0.074), but there were significant differences in the behavioral changes in the three groups under UPO stimulation (χ^2^ = 31.275, *p* = 0.000). In addition, pairwise comparisons showed that there were significant differences between the NC and MCI groups (χ^2^ = 7.209, *p* = 0.027) and between the NC and AD groups (χ^2^ = 23.794, *p* = 0.000). There was also a significant difference between the MCI and AD groups (χ^2^ = 15.520, *p* = 0.000).

## Discussion

By comparing olfactory fMRI and respiratory behavior results following stimulation by a PO and UPO, we found that the NCs and patients (MCI and AD) showed different patterns of activation in the POC and right F3O, and the changes in respiratory behavior among the NC, MCI, and AD groups were significantly different. These results indicated that stimulation by both the PO and UPO in olfactory fMRI can provide more information for the objective assessment of olfactory impairments associated with AD.

Neuropathological studies (24, 31–33) have confirmed the typical pathological changes in AD, such as neurofibrillary tangles and senile plaques, which appear in the POC at the early stage and then gradually involve other brain regions. This is the pathological basis of early olfactory dysfunction in AD patients. By using a PO (lavender) olfactory fMRI to investigate normal elderly individuals and AD patients, Wang et al. (17) found that the degree of POC activation in AD patients was significantly poorer than that in normal elderly individuals, and only a small amount of activation occurred under the stimulation conditions with the highest concentration. This result indicated that PO stimulation in fMRI can reflect the damaged state of the POC in AD patients. Regardless of whether the stimulation was by the PO or UPO, our study found that the number of activated voxels in the POC in the MCI and AD groups was significantly lower than that in the NC group, which indicated that UPO stimulation in olfactory fMRI can also reflect the damaged state of the POC in AD patients.

With PO (phenylethyl alcohol) stimulation in olfactory fMRI with healthy subjects, Poellinger et al. (34) found that a long period of continuous or repetitive odor stimulation can cause olfactory adaptation, which was manifested as changes in activation with increases in the time of odor stimulation: eventually, POC activation no longer increased and even decreased. In our study, the subjects were repeatedly stimulated with different concentrations of PO and UPO. The results showed that when the NC group was stimulated by the PO, as the concentration increased, the degree of POC activation first increased and then decreased, indicating that there was olfactory adaptation in the NC group. The degree of POC activation in the MCI and AD groups showed an increasing trend, indicating that olfactory adaptation had disappeared in the MCI and AD groups, which was in accordance with the results of Zhang et al. (18). The existence of olfactory adaptation may reflect that the NCs had a normal olfactory identification function. Decreases in this olfactory identification function in the MCI and AD patients led to a decrease or loss in olfactory adaptation. In our experiment, we found that olfactory adaptation also occurred when individuals were exposed to UPO (pyridine). However, this phenomenon was not exactly the same as that observed with the PO stimulation. With increases in UPO concentration, the level of POC activation in the NC group decreased, with activation levels being the highest with stimulation by the lowest concentration; the MCI and AD groups continued to show an increasing trend with increases in concentration. Compared with the findings on the PO, the NC group showed a higher activation level at low concentrations of the UPO, which may be related to the following factors: (1) the olfactory adaptation caused by the UPO was more sensitive than that caused by the PO; (2) the UPO can cause discomfort in human body. A higher concentration of UPO may cause subconscious respiratory behavior changes in NC subjects with normal olfaction, resulting in a decrease in inhaled gas. Moreover, with increases in UPO concentration, MCI and AD patients did not show olfactory adaptation, and also the number of activated voxels in the POC gradually increased; this may be the objective fMRI evidence in MCI and AD patients showing no distinction between fragrances and stenches.

Interestingly, when the PO and UPO were presented, the horizontal comparison between the NC, MCI, and AD groups showed a difference in the number of activated voxels in the POC, but a paired analysis of PO and UPO showed that the differences in the POC disappeared. We considered two possible reasons: first, the horizontal comparison was a direct intergroup comparison, and the paired analysis was used to subtract the two odor results within an individual, and then these were compared among the three groups. Second, because the main function of the POC is to perceive odor (35), regardless of whether a PO or UPO was used, the POC can be activated, and the paired analysis of the PO and UPO may have caused the difference to disappear.

It was reported that (21, 36–38) the orbitofrontal cortex was significantly activated during tasks involving familiarity, hedonism, and intensity of odor, which are considered to be the last step of odor identification. In addition, activation of the F3O has been associated with UPO stimulation (39). In this study, a paired analysis of the PO and UPO showed that the NC group had a statistically significant difference in the number of activated voxels in the right F3O, whereas there were no significant differences in this cerebral region in the MCI and AD groups, which might indicate that olfactory conduction and identification ability remained normal in the NC group and might be impaired in the MCI and AD groups. This study found for the first time differences in the right F3O between normal elderly individuals and patients with MCI and AD. This was the result of a paired analysis of PO and UPO stimulation. Therefore, the right F3O may be an important brain area for the evaluation of olfactory identification, and the use of both a PO and UPO can better analyze the functional state of this brain area. Remarkably, previous study (40–42) showed that the orbitofrontal cortex was the secondary olfactory center that received projections from the POC with olfactory information. Therefore, although we found a difference in the right F3O between the NC group and the patient groups, we still cannot determine whether it is caused directly by the invasion of hallmark pathological deposits in this region or due to the impaired olfactory function of the POC, leading to functional disconnection of the secondary olfactory cortices; further confirmation is needed.

Studies (17, 18) have found that there was a correlation between the number of activated voxels in the POC and neurocognitive test scores (MMSE and MoCA) with PO stimulation, indicating that the number of activated voxels in the POC can be used as an objective index to evaluate olfactory function. Our study showed that when the PO and UPO were presented, the number of activated voxels in the POC was correlated with MMSE and MoCA scores, and the correlation coefficients between PO and UPO were close. Moreover, we found that there was also a correlation with activation in the right F3O, where the correlation coefficient with the UPO was significantly higher than that with the PO. In addition, during UPO stimulation, the correlation coefficients between the number of activated voxels in this brain area and MMSE and MoCA scores (*r*_MMSE_ = 0.480, *r*_MoCA_ = 0.460) were significantly higher than the correlation coefficients (*r*_MMSE_ = 0.350, *r*_MoCA_ = 0.311) between the number of activated voxels in the POC and MMSE and MoCA scores, suggesting that activity in this brain region may be an important supplement to the objective evaluation of olfactory function in addition to the POC and that the combination of PO and UPO stimulation was better than PO alone.

Finally, we analyzed the respiratory behavior results recorded by the olfactory stimulator and found that during the inhalation of the PO, the number of short ventilation cycles was very small, and there were no significant differences in the respiratory behavior of the three groups. This may be because people are more receptive to a PO and do not subconsciously resist it. However, a small proportion of the subjects (9/73) took shallow breaths when inhaling a high concentration of PO. It was speculated that these subjects treated high concentrations of the PO as a UPO. In contrast, because the UPO was unacceptable, when the UPO was inhaled, most subjects in the NC group (21/25) had subconscious changes in ventilation, which manifested as reduced breathing amplitudes and shortened inhalation times. Most subjects in the MCI group (22/26) had similar changes in ventilation performance to those in the NC group. The difference was that most subjects in the NC group (17/25) showed this pattern of respiration at low concentrations, whereas only 9 of 26 participants in the MCI group did so at low concentrations. As we expected, no participants in the AD group (0/22) took short breaths during low concentration stimulation, which may be related to the increase in olfactory detection threshold, an impairment in olfactory identification ability, and an inability to effectively identify the UPO in AD patients. Therefore, it can be inferred that the changes in respiratory behavior after inhaled odors can reflect whether olfactory function is impaired, especially when the UPO is inhaled. As the respiratory changes after inhalation of the smell reflect the individual's subconscious behavior and lack subjective assumptions, it has more objectivity, which will play an important role in the clinical evaluation of an individual's olfactory function.

Through the application of both a PO and UPO, we not only identified differences between the NCs and MCI and AD patients from the imaging data but also showed the differences between the NCs and MCI and AD patients from the behavioral data, thereby providing more objective information for the assessment of olfactory impairment associated with AD.

There were also some limitations in our research. First, Philpott et al. (43) found that when receiving the same or similar odor stimulus, the olfactory adaptation clearance time was at least 15 min. In our study, the interval time of two odors was 25 min. Although the results of receiving two odor stimuli on two separate days may be more accurate, doing so would increase the burden on the patient and the complexity of the examination. The results from this study showed that differences between the NC group and the AD group can be obtained at an interval of 25 min. Second, we simply analyzed the changes in the respiratory amplitude recorded by the olfactory stimulator. We will further analyze the changes in the degree of respiratory amplitude, the length of the breath, and so on, to further and comprehensively evaluate olfactory function. Third, regarding the SPM statistics, our results did not pass the correction based on the cluster level, so we performed voxel-level tests (uncorrected *p* < 0.001, extent threshold = 70).

In short, with the application of both a PO and UPO in the context of fMRI, there were not only differences in brain area (POC and right F3O) activation between the NCs and the patients with AD and MCI but also significantly different respiratory behavior changes among NCs, MCI patients, and AD patients when stimulated by a low concentration of a UPO. Therefore, olfactory fMRI combined with a PO and UPO can provide more information for the objective assessment of hyposmia in AD based on imaging and behavior than a PO alone.

## Data Availability Statement

The original contributions presented in the study are included in the article/supplementary material, further inquiries can be directed to the corresponding author/s.

## Ethics Statement

The studies involving human participants were reviewed and approved by Tianjin Huanhu Hospital ethics committee. The patients/participants provided their written informed consent to participate in this study.

## Author Contributions

Material preparation and data collection were performed by QF, HL, HuiZ, YL, HuihZ, and YZ. Data analysis was performed by QF, GL, and TH. The first draft of the manuscript was written by QF. All authors commented on previous versions of the manuscript, read and approved the final manuscript, and contributed to the study conception and design.

## Conflict of Interest

The authors declare that the research was conducted in the absence of any commercial or financial relationships that could be construed as a potential conflict of interest.

## Publisher's Note

All claims expressed in this article are solely those of the authors and do not necessarily represent those of their affiliated organizations, or those of the publisher, the editors and the reviewers. Any product that may be evaluated in this article, or claim that may be made by its manufacturer, is not guaranteed or endorsed by the publisher.

## References

[1] Dubois B Feldman HH Jacova C Hampel H Molinuevo JL Blennow K. Advancing research diagnostic criteria for Alzheimer's disease: the IWG-2 criteria. Lancet Neurol. (2014) 13:614–29. 10.1016/S1474-4422(14)70090-0 24849862

[2] Ferreyra-Moyano H Barragan E. The olfactory system and Alzheimer's disease. Int J Neurosci. (1989) 49:157–97. 10.3109/00207458909084824 2700477

[3] Serby M Larson P Kalkstein D. The nature and course of olfactory deficits in Alzheimer's disease. Am J Psychiatry. (1991) 148:357–60. 10.1176/ajp.148.3.357 1992839

[4] Devanand DP Michaels-Marston KS Liu X Pelton GH Padilla M Marder K. Olfactory deficits in patients with mild cognitive impairment predict Alzheimer's disease at follow-up. Am J Psychiatry. (2000) 157:1399–405. 10.1176/appi.ajp.157.9.1399 10964854

[5] Tabert MH Manly JJ Liu X Pelton GH Rosenblum S Jacobs M. Neuropsychological prediction of conversion to Alzheimer disease in patients with mild cognitive impairment. Arch Gen Psychiatry. (2006) 63:916–24. 10.1001/archpsyc.63.8.916 16894068

[6] Jung HJ Shin IS Lee JE. Olfactory function in mild cognitive impairment and Alzheimer's disease: a meta-analysis. Laryngoscope. (2019) 129:362–9. 10.1002/lary.27399 30565695

[7] Doty RL Shaman P Dann M. Development of the University of Pennsylvania Smell Identification Test: a standardized microencapsulated test of olfactory function. Physiol Behav. (1984) 32:489–502. 6463130 10.1016/0031-9384(84)90269-5

[8] Djordjevic J Jones-Gotman M De Sousa K Chertkow H. Olfaction in patients with mild cognitive impairment and Alzheimer's disease. Neurobiol Aging. (2008) 29:693–706. 10.1016/j.neurobiolaging.2006.11.014 17207898

[9] Growdon ME Schultz AP Dagley AS Amariglio RE Hedden T Rentz DM. Odor identification and Alzheimer disease biomarkers in clinically normal elderly. Neurology. (2015) 84:2153–60. 10.1212/WNL.0000000000001614 25934852 PMC4451046

[10] Woodward MR Hafeez MU Qi Q Riaz A Benedict RHB Yan L. Odorant item specific olfactory identification deficit may differentiate Alzheimer disease from aging. Am J Geriatr Psychiatry. (2018) 26:835–46. 10.1016/j.jagp.2018.02.008 29858162 PMC6086738

[11] Kjelvik G Saltvedt I White LR Stenumgård P Sletvold O Engedal K. The brain structural and cognitive basis of odor identification deficits in mild cognitive impairment and Alzheimer's disease. BMC Neurol. (2014) 14:168. 10.1186/s12883-014-0168-1 25154749 PMC4236673

[12] Servello A Fioretti A Gualdi G Di Biasi C Pittalis A Sollaku S. Olfactory dysfunction, olfactory bulb volume and Alzheimer's disease: is there a correlation? A pilot study. J Alzheimers Dis. (2015) 48:395–402. 10.3233/JAD-150232 26402003

[13] Yu Q Guo P Li D Zuo L Lian T Yu S. Olfactory dysfunction and its relationship with clinical symptoms of Alzheimer disease. Aging Dis. (2018) 9:1084–95. 10.14336/AD.2018.0819 30574420 PMC6284764

[14] Kondo H Matsuda T Hashiba M Baba S. A study of the relationship between the TandT olfactometer and the University of Pennsylvania Smell Identification Test in a Japanese population. Am J Rhinol. (1998) 12:353–8. 10.2500/105065898780182390 9805536

[15] Kashibayashi T Takahashi R Fujita J Kamimura N Okutani F Kazui H. Correlation between regional brain volume and olfactory function in very mild amnestic patients. J Neurol Sci. (2020) 411:116686. 10.1016/j.jns.2020.116686 31972350

[16] Kashibayashi T Takahashi R Fujita J Fujito R Kamimura N Okutani F. Correlation between cerebral blood flow and olfactory function in mild cognitive impairment and Alzheimer's disease. Int J Geriatr Psychiatry. (2021) 36:1103–9. 10.1002/gps.5527 33751658

[17] Wang J Eslinger PJ Doty RL Zimmerman EK Grunfeld R Sun X. Olfactory deficit detected by fMRI in early Alzheimer's disease. Brain Res. (2010) 1357:184–94. 10.1016/j.brainres.2010.08.018 20709038 PMC3515873

[18] Zhang H Ji D Yin J Wang Z Zhou Y Ni H. Olfactory fMRI activation pattern across different concentrations changes in Alzheimer's disease. Front Neurosci. (2019) 13:786. 10.3389/fnins.2019.00786 31417348 PMC6682702

[19] Zelano C Bensafi M Porter J Mainland J Johnson B Bremner E. Attentional modulation in human primary olfactory cortex. Nat Neurosci. (2005) 8:114–20. 10.1038/nn1368 15608635

[20] Katata K Sakai N Doi K Kawamitsu H Fujii M Sugimura K. Functional MRI of regional brain responses to 'pleasant' and 'unpleasant' odors. Acta Otolaryngol Suppl. (2009) 562:85–90. 10.1080/00016480902915715 19848247

[21] Wu KN Tan BK Howard JD Conley DB Gottfried JA. Olfactory input is critical for sustaining odor quality codes in human orbitofrontal cortex. Nat Neurosci. (2012) 15:1313–9. 10.1038/nn.3186 22885850 PMC3431433

[22] Vasavada MM Wang J Eslinger PJ Gill DJ Sun X Karunanayaka P. Olfactory cortex degeneration in Alzheimer's disease and mild cognitive impairment. J Alzheimers Dis. (2015) 45:947–58. 10.3233/JAD-141947 25633674

[23] Vasavada MM Martinez B Wang J Eslinger PJ Gill DJ Sun X. Central olfactory dysfunction in Alzheimer's disease and mild cognitive impairment: a functional MRI study. J Alzheimers Dis. (2017) 59:359–68. 10.3233/JAD-170310 28671131

[24] Braak H Braak E. Neuropathological stageing of Alzheimer-related changes. Acta Neuropathol. (1991) 82:239–59. 1759558 10.1007/BF00308809

[25] Thal DR Rub U Orantes M Braak H. Phases of A beta-deposition in the human brain and its relevance for the development of AD. Neurology. (2002) 58:1791–800. 10.1212/WNL.58.12.1791 12084879

[26] Gray AJ Staples V Murren K Dhariwal A Bentham P. Olfactory identification is impaired in clinic-based patients with vascular dementia and senile dementia of Alzheimer type. Int J Geriatr Psychiatry. (2001) 16:513–7. 10.1002/gps.383 11376468

[27] Peters JM Hummel T Kratzsch T Lötsch J Skarke C Frölich L. Olfactory function in mild cognitive impairment and Alzheimer's disease: an investigation using psychophysical and electrophysiological techniques. Am J Psychiatry. (2003) 160:1995–2002. 10.1176/appi.ajp.160.11.1995 14594747

[28] Petersen RC. Mild cognitive impairment as a diagnostic entity. J Intern Med. (2004) 256:183–94. 10.1111/j.1365-2796.2004.01388.x 15324362

[29] Henkin RI Levy LM. Lateralization of brain activation to imagination and smell of odors using functional magnetic resonance imaging (fMRI): left hemispheric localization of pleasant and right hemispheric localization of unpleasant odors. J Comput Assist Tomogr. (2001) 25:493–514. 10.1097/00004728-200107000-00001 11473178

[30] Tzourio-Mazoyer N Landeau B Papathanassiou D Crivello F Etard O Delcroix N. Automated anatomical labeling of activations in SPM using a macroscopic anatomical parcellation of the MNI MRI single-subject brain. Neuroimage. (2002) 15:273–89. 10.1006/nimg.2001.0978 11771995

[31] Price JL Davis PB Morris JC White DL. The distribution of tangles, plaques and related immunohistochemical markers in healthy aging and Alzheimer's disease. Neurobiol Aging. (1991) 12:295–312. 10.1016/0197-4580(91)90006-6 1961359

[32] Attems J Jellinger KA. Olfactory tau pathology in Alzheimer disease and mild cognitive impairment. Clin Neuropathol. (2006) 25:265–71. 10.1080/02699200500245798 17140156

[33] Wilson RS Arnold SE Schneider JA Tang Y Bennett DA. The relationship between cerebral Alzheimer's disease pathology and odour identification in old age. J Neurol Neurosurg Psychiatry. (2007) 78:30–5. 10.1136/jnnp.2006.099721 17012338 PMC2117790

[34] Poellinger A Thomas R Lio P Lee A Makris N Rosen BR. Activation and habituation in olfaction–an fMRI study. Neuroimage. (2001) 13:547–60. 10.1006/nimg.2000.0713 11305885

[35] Bensafi M. The role of the piriform cortex in human olfactory perception: insights from functional neuroimaging studies. Chemosens Percept. (2012) 5:4–10. 10.1007/s12078-011-9110-8

[36] Gottfried JA O'Doherty J Dolan RJ. Appetitive and aversive olfactory learning in humans studied using event-related functional magnetic resonance imaging. J Neurosci. (2002) 22:10829–37. 10.1523/JNEUROSCI.22-24-10829.2002 12486176 PMC6758414

[37] Kringelbach ML O'Doherty J Rolls ET Andrews C. Activation of the human orbitofrontal cortex to a liquid food stimulus is correlated with its subjective pleasantness. Cereb Cortex. (2003) 13:1064–71. 10.1093/cercor/13.10.1064 12967923

[38] Plailly J Bensafi M Pachot-Clouard M Delon-Martin C Kareken DA Rouby C. Involvement of right piriform cortex in olfactory familiarity judgments. Neuroimage. (2005) 24:1032–41. 10.1016/j.neuroimage.2004.10.028 15670680

[39] Gottfried JA Deichmann R Winston JS Dolan RJ. Functional heterogeneity in human olfactory cortex: an event-related functional magnetic resonance imaging study. J Neurosci. (2002) 22:10819–28. 10.1523/JNEUROSCI.22-24-10819.2002 12486175 PMC6758422

[40] Savic I Gulyas B Larsson M Roland P. Olfactory functions are mediated by parallel and hierarchical processing. Neuron. (2000) 26:735–45. 10.1016/S0896-6273(00)81209-X 10896168

[41] Lundström JN Boesveldt S Albrecht J. Central processing of the chemical senses: an overview. ACS Chem Neurosci. (2011) 2:5–16. 10.1021/cn1000843 21503268 PMC3077578

[42] Wang J Sun X Yang QX. Early aging effect on the function of the human central olfactory system. J Gerontol A Biol Sci Med Sci. (2017) 72:1007–14. 10.1093/gerona/glw104 27289103 PMC5861935

[43] Philpott CM Wolstenholme CR Goodenough PC Clark A Murty GE. Olfactory clearance: what time is needed in clinical practice? J Laryngol Otol. (2008) 122:912–7. 10.1017/S0022215107000977 18036276

